# Prognostic and Clinicopathological Significance of MiR-155 in Breast Cancer: A Systematic Review

**DOI:** 10.3390/ijms21165834

**Published:** 2020-08-14

**Authors:** Anna Maria Grimaldi, Silvia Nuzzo, Gerolama Condorelli, Marco Salvatore, Mariarosaria Incoronato

**Affiliations:** 1 IRCCS SDN, Via Emanuele Gianturco 113, 80143 Naples, Italy; annamaria.grimaldi@synlab.it (A.M.G.); silvia.nuzzo@synlab.it (S.N.); direzionescientifica@sdn-napoli.it (M.S.); 2 Department of Molecular Medicine and Medical Biotechnology, “Federico II” University of Naples, Via Pansini 5, 80131 Naples, Italy; gecondor@unina.it; 3 IRCCS Neuromed, Istituto Neurologico Mediterraneo, 86077 Pozzilli, Italy

**Keywords:** microRNA, miR-155, breast cancer, prognosis, diagnosis, tissue, circulating

## Abstract

There is an unmet need for novel non-invasive prognostic molecular tumour markers for breast cancer (BC). Accumulating evidence shows that miR-155 plays a pivotal role in tumorigenesis. Generally, miR-155 is considered an oncogenic miRNA promoting tumour growth, angiogenesis and aggressiveness of BC. Therefore, many researchers have focused on its *use* as a *prognostic* biomarker and therapeutic target. However, its prognostic value for BC patients remains controversial. To address this issue, the present systematic review aims to summarize the available evidence and give a picture of a prognostic significance of miR-155 in BC pathology. All eligible studies were searched on PubMed and EMBASE databases through various search strategies. Starting from 289 potential eligible records, data were examined from 28 studies, comparing tissue and circulating miR-155 expression levels with clinicopathological features and survival rates in BC patients. We discuss the pitfalls and challenges that need to be assessed to understand the power of miR-155 to respond to real clinical needs, highlighting the consistency, robustness or lack of results obtained to sate in translating this molecule to clinical practice. Our paper suggests that the prognostic role of miR-155 in the management of BC needs to be further verified.

## 1. Introduction

Breast cancer (BC) represents one of the most frequent malignant neoplasms in women worldwide, and its mortality rate is still increasing, due to population growth and ageing [1]. Worldwide BC mortality trends for the current year are estimated to be 656 thousand, with an incidence of about 2.18 million women [2]. This suggests that, due to its heterogeneity, several types of BC could be more aggressive, leading to a high female mortality rate. Once patients receive a diagnosis of BC, the first step is to understand their prognosis for optimal management, thus avoiding overtreatment of nonaggressive disease or undertreatment of aggressive forms. For this purpose, clinical criteria such as tumour size, tumour grade, number of lymph node (LN) metastases, patient age and patient morbidity status are used. Nevertheless, in the last 20 years, several studies have focused on the identification of new prognostic biomarkers in tissues and biofluids (plasma, serum, urine) to demonstrate their clinical translatability.

MicroRNAs (miRNAs) are a class of nucleic acid-based molecules approximately 19 to 24 nucleotides long and regulate gene expression at the post-transcriptional level [3]. MiRNA interactions with target sites located on 3’ untranslated region of the mRNAs induce degradation or translation inhibition of the targets [4]. MiRNAs have been found involved in multiple critical biological processes, and their deregulation has been associated with a wide range of human pathologies, including cancer [5]. Being able to regulate the expression of oncogenes and tumour-suppressors, they have been recognized as oncogenes and tumour suppressors, and their deregulation has been proposed for diagnostic and prognostic purposes [6]. To date, miRNA biomarkers are not currently used in clinical practice, because their translation from the lab into validated diagnostic tests represents a major challenge. Among the miRNAs found to be deregulated in BC, miR-21 and miR-155 have been identified as the most frequently BC-associated, although they are the most non-specific for diagnostic purposes [7]. Moreover, a recent meta-analysis has reported that miR-21 has a strongly predictive role and may be applied as a general prognostic marker of BC [8]. Irrespective of its diagnostic role, several studies have evaluated the prognostic role of miR-155 in BC: in a recent article, authors correlated the ability of a miRNA signature including miR-155 for prognostic purposes only in Triple Negative BC (TNBC) [9]. Since the prognostic value of miR-155 expression in BC has not yet been systematically analysed for all BC subtypes, we considered it useful to conduct a systematic review on the prognostic role of miR-155 in BC. The purpose of this study was to examine the clinical studies conducted to date on miR-155 as a tissue or peripheral blood BC biomarker. We wanted to understand its ability to respond to real clinical needs, highlighting the consistency, strength or lack of results obtained to date for addressing miR-155 use for prognostic purposes.

## 2. Results

### 2.1. Literature Search Results

**Figure 1:**
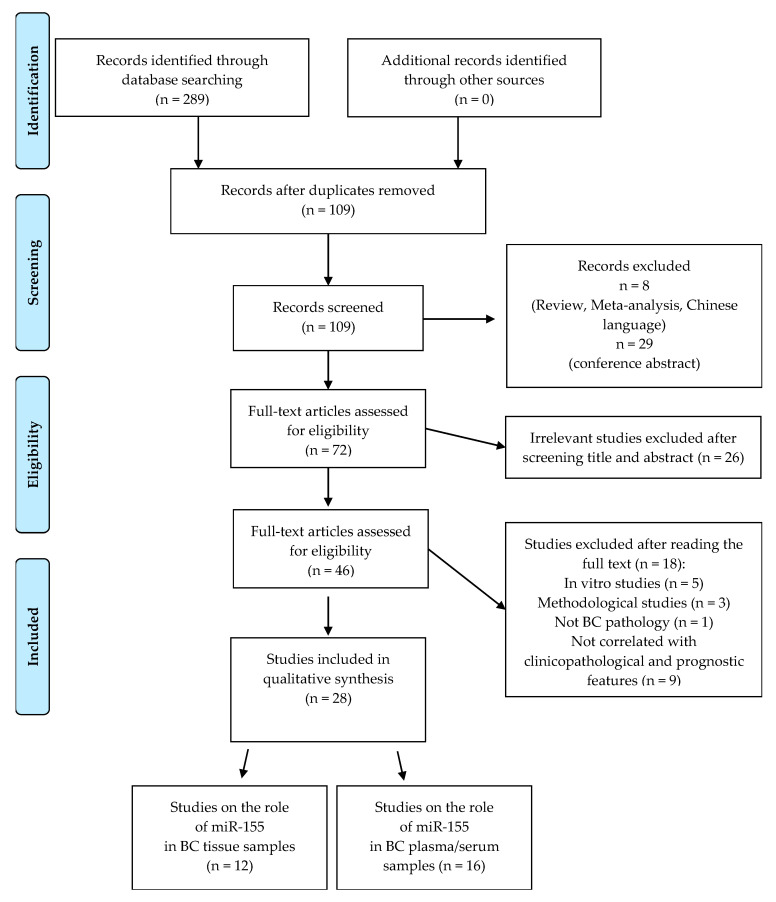
Flowchart for the strategy searches and selection processes.

Following our search strategy, a total of 289 potential eligible records related to the prognostic role of miR-155 in BC were extracted from PubMed and EMBASE public databases. Figure 1 shows the detailed selection of the articles. Specifically, 180 duplicates were deleted, leaving 109 records, of which 37 were excluded because they were non-research articles or non-English literature publications. From the remaining 72 articles, 26 were excluded because they were irrelevant after screening the title and abstract. The remaining 46 eligible articles were all downloaded to obtain valid information, and 18 studies were eliminated due to lack of available prognostic-related data. Thus, this systematic review is based on 28 eligible studies to assess the prognostic role of miR-155 in BC tissue and plasma/serum. In particular, we classified these selected studies in two groups: the first included 12 studies that analysed the prognostic role of miR-155 in tissues; the second contained a total of 16 studies, 11 of which analysed the prognostic role of circulating miR-155 and five of which analysed tissue and serum/plasma samples, even if with a major focus on the prognostic role in blood.

The main characteristics of each study are detailed in Table 1 and Table 2. Overall, the 28 original articles included were published between 2010 and 2020 and had a sample size ranging from 30 to 295, with a total of 3752 samples; 20 studies included miR-155 in a shortlist of two or more deregulated miRNAs, the remaining eight papers assessing only miR-155. Among all selected papers, 16 studies exclusively correlated miR-155 expression with patient-related prognostic factors (age, race, reproductive factor, family history, high-risk gene mutations) or/and tumour-related prognostic factors (tumour size, grade, differentiation, stage, involvement of axillary LN). Furthermore, 12 studies gave more exhaustive information, also using risk assessment models such as disease-free survival (DFS), disease metastasis-free survival (DMFS) and overall survival (OS). Regarding miRNA detection methods, Real-Time Quantitative Reverse Transcription PCR (RT-qPCR) was used in 26 studies and NanoString analysis in two studies.

### 2.2. Prognostic Value of MiR-155 in Tissues of BC Patients

Among the 12 selected studies assessing the prognostic value of tissue miR-155 in BC patients (Table 1), three of them pursued this goal exclusively through the association of miR-155 expression levels with patient-related prognostic factors (age, race, reproductive factor, family history, high-risk gene mutations) and tumour-related prognostic factors (receptors status, tumour size, grade, differentiation, stage, involvement of axillary LN), while the remaining studies built also prognostic models (DFS, DMFS and OS) and/or evaluated its response to therapy.

#### 2.2.1. Tissue MiR-155 Versus Patient- and Tumour-Related Prognostic Factors

Most of the selected studies agree with the positive correlation between miR-155 overexpression and tumour grade [10,11], Tumour Node Metastasis stage (TNM) [10,12,19] and LN positivity [10,12,19]. Conversely, some discordances emerge about the miR-155 correlation with hormonal receptor status (ER, PR, and HER2). Specifically, Hafez et al. [11], Chen et al. [10] and Jang et al. [17] did not find any association between miR-155 expression and ER/PR status. Instead, Zhen et al. [12] reported that high expression levels of miR-155 were associated with positive ER/PR status and low miR-155 expression correlated with negative ER/PR status. Although Iyeveleva et al. [13] and Gwak et al. [14] found that miR-155 correlated with ER/PR status, their results opposed [12] because both authors found that tissue levels of miR-155 were inversely correlated with the receptors status. Only two studies evaluated the association between miR-155 expression and HER2 status and their findings resulted completely discordant: Zhen et al. [12] did not find any association between miR-155 and HER2, whereas Gwak et al. [14] found that lower expression of miR-155 correlated with HER2-positive status. In the same way, Zhen et al. [12] did not find any association between miR-155 and tumour subtypes, whereas Gwak et al. [14] found that the tissue miR-155 expression was significantly higher in luminal A and TNBC subtypes compared to luminal B, luminal B/HER2 positive and HER2 positive subtypes. Correlation studies between tissue levels of miR-155 and tumour size were also somewhat in disagreement. In fact, Hafez et al. [11] found that tissue miR-155 expression was significantly associated with tumour size, whereas no correlation was found by [10,12]. The association of miR-155 and Ki-67 was explored only in one study, demonstrating that these two molecules were positively correlated [12]. Finally, the results of Jang et al. [17] were discouraging, and, focusing exclusively on TNBC patient cohort, did not find any association of miR-155 expression with the aforementioned clinicopathological parameters. In the same way, no significant correlations were found between miR-155 expression and patient-related prognostic factors [10,12].

#### 2.2.2. Tissue miR-155 Versus Prognostic Models and Response to Therapy

Most of the selected studies associated miR-155 level with poor prognosis in BC patients. Chen et al. proposed miR-155 as a negative prognostic marker using a risk model of DFS and OS [10]. In details, patients with high miR-155 expression showed significantly lower DFS and OS rates. In the same study, the univariate analysis demonstrated that miR-155, tumour grade, TNM stage, LN metastases and c-erbB-2 expression were all significantly associated with poor OS, whereas the multivariate analysis identified miR-155 and LN metastases as independent factors for predicting prognosis in BC patients. Similarly, Zhang et al. [19] reported a close association between tissue miR-155 overexpression and shorter OS. Differently, Gasparini et al. asserted a protective role for miR-155 in TNBC [15,16]. They tested the diagnostic and prognostic capability of a signature of four miRNAs (miR-155, miR-493, miR-30e and miR-27a) [15]. This signature could subdivide TNBC patients into high- and low-risk groups and two subtypes: basal-like TN (Coral basal: negative for ER, PR, HER2, and positive for CK5/6 and/or EGFR) and five negatives (negative for ER, PR, HER2, CK5/6 and EGFR). OS analysis indicated a significant association between the expression of the 4-miRNA signature and survival in TNBC, with up-regulation of miR-155 that correlated with a better patient outcome. Moreover, the designed miRNA signature predicted patient outcomes based on the two most commonly used chemotherapy regimens (anthracycline or anthracycline plus taxanes). In addition, in [16] the authors reaffirmed, in a cohort of 93 TNBC treated with chemotherapy plus radiation or exclusively with radiation, that miR-155 is positively correlated with OS.

Raychaudhuri et al. [18] evaluated whether the relative expression of 10 miRNAs, including miR-155, could be used for predicting neoadjuvant chemotherapeutic response and if the differential expression in residual tumour after treatment could allow prognostic stratification of non-responding patients. In particular, they found a significant decrease in miR-155 expression in residual tumour after chemotherapy, and its low intra-tumoural expression at baseline was significantly associated with longer DFS and OS. Differently, Jang et al. [17], in TNBC patients, did not find any association between miR-155 expression and chemotherapeutic regiments and the high expression of miR-155 was associated with a longer DMFS, but not with DFS. Finally, three of the 12 selected studies [14,20,21] did not find any association among miR-155 expression with prognostic assessment models.

#### 2.2.3. Tissue MiR-155 vs. Molecular Tumour Markers

In two of the 12 selected studies, miR-155 expression was correlated with oncogenic markers. Hafez et al. [11] reported that the up-regulation of miR-155 was associated with the down-regulation of anti-angiogenic activity of TIMP1, TMP1 and TIMP3 genes, the up-regulation of genes involved in tumour invasion and metastasis (MMP2 and MMP9) and with upregulation of VEGF, known to be linked to angiogenesis. Moreover, Jang et al. [17] found that miR-155 expression was inversely correlated with several markers involved in the epithelial–mesenchymal transition process, including SMA, osteonectin and CD146.

### 2.3. Prognostic Value of MiR-155 in Plasma/Serum of BC Patients

Most of the eligible studies selected (Table 2) assessed the prognostic value of circulating miR-155, exclusively correlating its expression with patient-related prognostic factors and tumour-related prognostic factors, while just three studies also performed prognostic models by monitoring patients during follow-up. In these 16 studies, circulating miR-155 was screened in serum or plasma, 5 of them analysing miR-155 expression simultaneously in blood and tissue. Besides, among the 16 studies, 13 of them analysed miR-155 deregulation together with other circulating miRNAs.

#### 2.3.1. Circulating MiR-155 Versus Patient- and Tumour-Related Prognostic Factors

The studies focusing on the deregulation of miR-155 in blood samples of BC patients with prognostic factors provide heterogeneous results. Regarding tumour size, Answar et al. [37] assessed the relationship between circulating miR-155 and tumour size, finding a significantly higher miR level in BC patients with tumours larger than 5 cm, whereas Jukovicova et al. [26] and Soleimanpour et al. [31] found no significant association. Concerning tumour hormonal status, Swellam et al. [35] and Jurkovicova et al. [26] did not find any significant correlations among serum and plasma miR-155 and ER/PR status, respectively. On the contrary, Wang et al. [22] found that serum miR-155 overexpression correlated with ER/PR-negative status. Similar discrepancies were found when other tumour parameters were assessed: Swellam et al. [35] and Wang et al. [22] found that serum miR-155 correlated with grade, whereas Jurkovicova et al. [26] and Anwar et al. [37] did not find this correlation. Regarding the TNM stage, five studies [25,27,29,30,35] reported that increased expression of circulating miR-155 was significantly associated with advanced stage, whereas the other four studies [23,30,31,37] did not find any correlations. Similarly, patchy results were published considering LN metastases. In detail, Liu et al. [27], Huang et al. [29] and Lukianova et al. [32] found that increased expression of serum miR-155 was significantly associated with LN metastases, whereas some other studies [26,30,31,35] reported no relationship between miR-155 expression and LN involvement. In single studies, circulating miR-155 was significantly associated with Ki-67 [26] and with TNBC [32]. Concerning the miR-155 association with tumour BC subtype, Lukiana et al. found a significant correlation with TNBC subtypes, but opposing this were the results obtained by Anwar and colleagues [37]. Nevertheless, Zaleski et al. [28] tested the diagnostic and prognostic relevance of miR-155 in serum of 103 BC subjects and did not find any clinical implications. Considering the patient-related prognostic factors, the only significant correlations were found versus age [22], menarche age of < 13 years, many artificial abortions, high body mass index (BMI) or a family history of BC [25].

Finally, Fortis and colleagues [34] evaluated the expression of six circulating miRNAs (miR-155, -16, -21, -23α, -146α and -181α) in non-metastatic BC patients and put it in a relationship with blood cytokines, tumour-immune-cell infiltration and clinicopathological features to reflect the clinical outcomes. The authors identified three different miRNA clusters (low, moderate and high expression levels) able to identify three distinct groups of BC patients featured by their clinicopathological and immune-related characteristics, with possible different clinical prognosis.

#### 2.3.2. Circulating MiR-155 vs. Prognostic Models and Response to Therapy

Prognostic rates, such as OS and DFS, were poorly investigated in association with circulating miR-155 expression level. Song et al. [36] reported that high expression levels of plasmatic miR-155 in TNBC patients significantly correlated with poor survival. In accordance with this finding, Guo and collaborators [25] performing Kaplan–Meier analysis, reported that subjects with low serum miR-155 (cutoff < 1.24 U/mL) survived significantly longer than subjects with high serum levels. Besides, independently of whether patients received treatment or not, patients with low miR-155 expression showed significantly longer survival. Similarly, Swellam found that patients with higher levels of circulating miRNA-155 showed worse DFS, but no significant result was reported for OS [35]. Moreover, Huang et al. [29] observed a significant decrease in miR-155 expression after treatment with the chemoterapic agent vinorelbune, suggesting miR-155 as a potential monitoring marker of clinical outcome. Similar results were reported also by Khalighfard et al. [30] and Anwar and colleagues [37]. They proposed miR-155 as a biomarker for monitoring treatment response as its plasma levels were significantly downregulated after treatments. Differently from previous findings, no predictive value for miR-155 was found after neoadjuvant chemotherapy treatment [27]. Finally, recently, Rodriguez-Martinez and colleagues [33] proposed the use of exosomal miRNAs and circulating tumour cells as diagnostic and predictive biomarkers in BC patients under neoadjuvant treatment. They demonstrated a positive correlation between miR-155 expression and the number of circulating tumour cells (CTCs) ≥ 3CTCs/10 mL at diagnosis.

### 2.4. HMDD Database

**Figure 2:**
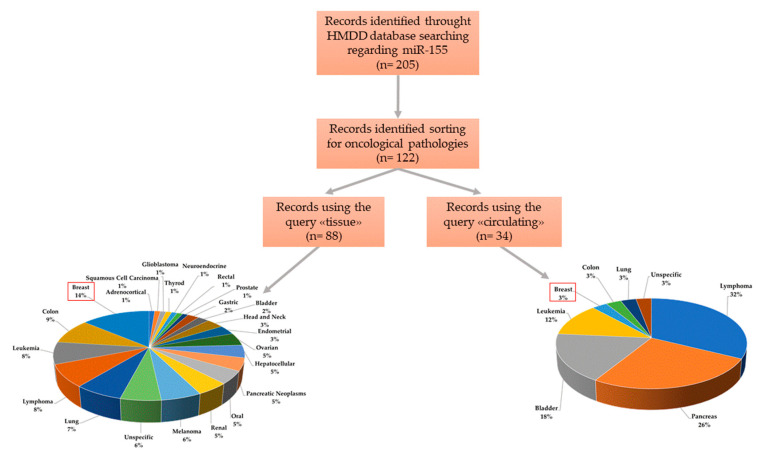
Overview of disease specificity of miR-155 in different human oncological pathologies.

We interrogated the Human miRNA disease database (HMDD 3.2v) [38], experimentally supported and manually curated, that collects data of miRNAs associated with the development and progression of a broad range of diseases. We first assessed the engagement of miR-155 in human diseases by the “disease spectrum width” (DSW), a parameter calculated as the ratio between the number of diseases associated with a miRNA and the total number of diseases [39]. The high score calculated for miR-155 (DSW = 0.281) was second only to miR-21, indicating a great number of pathologies in which miR-155 seems to be involved. Then, sorting only for miR-155, we identified 205 studies specifically associated with it (Figure 2). Among them, about 60% (n = 122) were oncological pathologies, divided into 88 studies on tissues and 34 on blood. As reported in Figure 2, 14% of selected studies reported miR-155 as a tissue-deregulated miRNA associated with BC, whereas 3% of studies associated its circulating deregulation with BC. Although the non-specificity of miR-155 in human cancers was previously found in our study [7], the highest number of studies associating miR-155 with BC (14%) suggests its potential role as a tissue biomarker. However, circulating miR-155 seems to have a better role as a biomarker of pancreatic cancer (26%) than of BC (3%).

## 3. Discussion and Conclusions

Here, we have analysed the potential prognostic value of miR-155 in BC. MiRNAs are useful tools for diagnostic and prognostic purposes in several diseases [40]. In recent years, many studies have investigated the clinical value of miRNAs in BC [7,41], a worldwide public health problem that poses significant challenges in terms of disease management. To date, miR-155 represents one of the most studied miRNAs in different human pathologies, including BC. In fact, miR-155 regulates cancer progression [42] and it is involved in drug resistance in BC, which is a reason why miR-155 antisense oligonucleotides (antimiR-155) could represent an interesting therapeutic approach [43]. Ti date, antimiR-155 approaches have been developed and tested in vitro but the most effective way to deliver it in vivo remains to be solved, therefore its application for therapeutic purposes is still premature.

Many researchers have focused on the possibility of utilizing miR-155 as a biomarker for diagnostic and prognostic purposes in BC. Nevertheless, the benefits of using this miRNA as a prognosis marker remain unclear. We have carried out a systematic review of all studies in which miR-155 seems to provide promising results as a prognostic molecule. In this qualitative analysis, starting from 289 identified records, we selected a total of 28 studies that revealed the potential role of miR-155 for prognostic purposes in BC. Because these studies were highly heterogeneous and only a few of them performed survival analysis, the data have been somewhat difficult to aggregate. In most of them, authors performed correlation analyses of miR-155 expression with prognostic patient-related factors (age, menarche, BMI, family history and abortion) and with prognostic tumour-related factors (ER, PR, HER2, grade, Ki-67, TNM stage, LN metastases and tumour size). Regrettably, however, not in every study was OS or DFS calculated, maybe due to a lack of follow-up data, making the clinical application of this miR doubtful. However, about 90% of these studies correlated increasing tissue and/or circulating miR-155 with poor prognosis, whereas only in three of them was miR-155 up-regulation linked to better prognosis [15,16,17]; authors proposed a protective role for miR-155, distancing it from its traditional oncomiR role. To aggravate this uncertainty, four studies determined no significant correlation between miR-155 deregulation and prognosis [20,23,28,31].

More concordant results have correlated miR-155 expression with therapeutic response [24,29,37]. In fact, in those studies, the authors found that circulating miR-155 decreased after treatment (surgical or pharmacological).

Summarizing, our systematic review has elucidated that miR-155, although promising it is still far from being an effective biomarker for predicting prognosis of BC patients. Nevertheless, to date, only one on-going clinical trial (NCT02605512, https://clinicaltrials.gov/), started on 2015, involves the miR-155 as a biomarker of cardiotoxicity induced by RAdioTherapy in BC, but results are not yet available. Unlike what was expected, the selected studies did not provide sufficient information to give a consistent answer to our research question. In this framework, the differences in sample size, cohort demographics, inhomogeneous subtype distributions and follow-up time-table could account for the general lack of strength of results. Thus, further, large-scale studies with better design and more comprehensive follow-up data support will need to be conducted to clarify the prognostic value of miR-155 in BC. Overall, this work aims to highlight the weaknesses that need to be overcome to validate the prognostic role of miR-155 in BC, as its use in clinical practice appears still premature. Although miRNA application in clinical practice is lacking to date, experimental results could make it possible to use these molecules for prognostic purposes. However, despite the interesting intrinsic characteristics of miRNAs, several challenges for their clinical application in the management of oncological diseases still needs to be overcome.

## 4. Materials and Methods

### 4.1. Search Strategy

A systematic literature search was performed by three authors independently (AMG, SN and MI) on PubMed and EMBASE databases. We used the following search strategies: (miR-155, microRNA-155, miRNA-155, miR155) and breast cancer and (prognosis or prognostic or tissue or circulating or serum or plasma) (key terms listed in Table S1). Besides, we also did a manual search by using the reference lists of important articles published to include all eligible studies.

### 4.2. Selection Criteria

Three researchers (AMG, SN and MI) independently, after having screened identified studies for titles and abstracts, included or excluded articles basing on the following criteria. Inclusion criteria: (1) prognostic role of miR-155 in breast tissues and/or blood samples of BC patients; (2) original articles; (3) English language. Exclusion criteria: (1) letters, case reports, reviews, conference abstracts; (2) non-English papers; (3) studies performed on immortalized BC cell lines; (4) methodological studies; (5) studies not focused on BC. The discrepancies between the three reviewers were resolved by consensus or by discussion with a fourth researcher (GC).

### 4.3. Data Extraction and Collection

After the selection procedure, data that met the inclusion criteria were summarized into a customized Excel spreadsheet database by one investigator (AMG) and verified by a second and third (SI and MI). For each study, the following characteristics were collected: publication year; country of the population enrolled; cohort study; sample; method; miRNAs; miR-155 expression; prognosis; significantly associated parameters; survival analysis; reference.

### 4.4. Planning and Conducting the Review

The articles were classified based on the sample type used in studies: tissue and/or plasma/serum samples. This systematic review was conducted following the Preferred Reporting Items for Systematic Reviews and Meta-Analyses (PRISMA) guidelines [44] (See Table S2 for PRISMA Checklist).

### 4.5. HMDD Database

We integrated our systematic review data with the open web-based resource Human miRNA disease database (HMDD v3.2) [38]. We assessed the engagement of miR-155 in human diseases by the “disease spectrum width” (DSW), provided as the ratio between the number of diseases associated with miRNA and the total number of diseases [39]. Then, we downloaded all data and selected in the column “miR” the query “miR-155” and in the column “categories” the queries “circulation_biomarker” and “tissue_expression”. These data were sorted according to the spectrum of the oncological pathologies in the column “diseases”.

## Figures and Tables

**Table 1:** Comprehensive characteristics of selected studies and association between tissue miR-155 and prognostic characteristics in breast cancer (BC).

Year	Country	Cohort Study	Sample	Method	miRNAs	miR-155 Expression	Prognosis	Significantly Associated Parameters	Survival Analysis	Reference
2012	China	92 BC	Frozen T	RT-qPCR	miR-155	↑	Poor	Tumour grade, LN metastases, TNM, DFS, OS	KM curve, multivariate, univariate	[10]
2012	Egypt	40 BC, 40 CNT	Frozen T	RT-qPCR	miR-10b, -21, -155, -373	↑	Poor	tumour size, tumour grade, Metastases-related genes	np	[11]
2012	China	42 BC, 42 CNT	Frozen T	RT-qPCR	miR-155	↑	Poor	TNM, LN metastases, Ki-67, ER/PR+	np	[12]
2012	NA	120 BC	T	RT-qPCR	miR-155, -10b, -21, -31	↑	Poor	ER−	np	[13]
2014	Korea	295 BC	FFPE-T	RT-qPCR	miR-9, -155, -200a	↑	Poor	ER/PR−, Her2−, subtype	KM curve	[14]
2014	USA	173 BC	FFPE-T	Nanostring	miR-27a, -30e, -155, -493	↑	Better	Better outcome	KM curve, univariate	[15]
2014	USA	173 BC	FFPE-T	Nanostring	miR-155	↑	Better	OS	KM curve	[16]
2017	Korea	190 BC	FFPE-T	qRT-qPCR	miR-9, -155	↑	Better	EMT markers, DMFS	KM curve, multivariate, univariate	[17]
2017	Germany	106 BC	FFPE-T	RT-qPCR	miR-7, -21, -29a, -29b, -34a, -125b, -155, -200c, -340, -451	↑	Poor	DFS, OS	KM curve, multivariate	[18]
2019	China	100 BC, 28 CNT	Fresh T	RT-qPCR	miR-155	↑	Poor	LN metastases, TNM, OS	KM curve	[19]
2019	Japan	291 BC	FFPE-T	RT-qPCR	miR-155	↑	ns	ns	KM curve	[20]
2020	Iran	15 BC, 15 CNT	Frozen T	RT-qPCR	miR-27b, -29a, -155	↑	ns	prevascular invasion	KM curve	[21]

Legend: ↑ = up-regulated; ns = not statistically significant; np = not performed; KM curve = Kaplan Meier curve; BC = breast cancer; CNT = control patients; T = tissue; FFPE = Formalin-Fixed Paraffin-Embedded; TNM = Tumour Node Metastasis stage; LN = Lymph node, EMT = epithelial–mesenchymal transition.

**Table 2:** Comprehensive characteristics of selected studies and association between circulating miR-155 and prognostic characteristics in BC.

Year	Country	Cohort Study	Sample	Method	miRNAs	miR-155 Expression	Prognosis	Significantly Associated Parameters	Survival Analysis	Reference
2010	China	68 BC, 40 CNT	S + T	RT-qPCR	miR-21, -106a, -126, -155, -199a, -335	↑	Poor	Age, tumour grade, ER−, PR−	np	[22]
2013	Mexico	61 BC, 10 CNT	S + T	RT-qPCR	miR-10b, -21, -125b, -145, -155, -191, -382	↑	-	ns	np	[23]
2014	Finland	63 BC, 21 CNT	S	RT-qPCR	miR-155, -19a, -24, -181b	↑	Poor	treatment, risk	multivariate	[24]
2016	China	148 BC, 142 CNT	S	RT-qPCR	miR-155	↑	Poor	Menarche, abortions, BMI, family history, TNM stage, OS	KM curve	[25]
2017	Slovakia	137 BC, 11 CNT	P	RT-qPCR	miR-17, -18a, -19a, -20a, -21, -27a, -155	ns	Poor	Ki-67	np	[26]
2017	China	118 BC, 30 CNT	S	RT-qPCR	miR-155, -19a, -21, -125b, -155, -205, -373	↑	Poor	Stage, LN metastases	np	[27]
2018	Germany	55 BC, 20 BL, 28 CNT	S	RT-qPCR	miR-21, -34a, -92a, -155, -222, let-7c	ns	ns	ns	np	[28]
2018	China	158 BC, 107 CNT	S	RT-qPCR	miR-155, -574-5p, let-7a	↑	Poor	TNM stage, treatment, LN metastases	np	[29]
2018	Iran	30 BC, 10 CNT	P + T	RT-qPCR	miR-10b, -21, -155, Let-7a	↑	Poor	TNM stage, LN metastases, treatment	np	[30]
2019	Iran	30 BC, 25 CNT	P + T	RT-qPCR	miR-21, -155	↑	-	ns	np	[31]
2019	Ukraine	89 BC, 53 BL, 14 CNT	S + T	RT-qPCR	miR-155, -205, -320a	↑	Poor	LN metastases, TN subtype	np	[32]
2019	Spain	53 BC, 8 CNT	S	RT-qPCR	miR-21, -105, -155, -222, -221	↑	Poor	CTC	np	[33]
2019	Greece	48 BC	S	RT-qPCR	miR-16, -21, -23α, -146α, -155, -181α	ns	Poor	Worse outcome	np	[34]
2019	Egypt	96 BC, 47 BL, 39 CNT	S	RT-qPCR	miR-21, -126, -155	↑	Poor	TNM stage, grade, DFS	KM curve	[35]
2019	China	64 BC, 58 CNT	P	RT-qPCR	miR-155	↑	Poor	OS	KM curve	[36]
2020	Indonesia	102 BC, 15 CNT	P	RT-qPCR	miR-155	↑	Poor	Age, tumour size, treatment	np	[37]

Legend: ↑ = up-regulated; ns = not statistically significant; np = not performed, KM curve = Kaplan Meier curve; BC = breast cancer; CNT = control; BL = benign lesion; S = Serum; P = Plasma; T = tissue; TN = triple negative.

## Supplementary Materials

The following are available online at https://www.mdpi.com/1422-0067/21/16/5834/s1, Table S1: Key terms used in literature search, Table S2: PRISMA Checklist.

## Abbreviations

BC	Breast cancer
TNBC	Triple-negative breast cancer
OS	Overall survival
DFS	Disease-free survival
DMFS	Metastasis disease-free survival
RT-qlPCR	Quantitative Reverse Transcription Polymerase Chain Reaction
ER	Estrogen receptor
HER2	Human epidermal growth factor receptor 2
PR	Progesterone receptor
LN	Lymph node
CTCs	Circulating tumour cells
TNM	Tumour, Node, Metastasis
